# Follow-up of Ebola Patient, 2014–2015

**DOI:** 10.3201/eid2204.151405

**Published:** 2016-04

**Authors:** Venkatesh Srinivas, Pragya Yadav, Veena Mittal, Gouri Chaubal, Mala Chhabra, Sanjay Mattoo, Arvind Rai, Devendra T. Mourya

**Affiliations:** National Centre for Disease Control, New Delhi, India (S. Venkatesh, V. Mittal, M. Chhabra, A. Rai);; National Institute of Virology, Pune, India (P. Yadav, G. Chaubal, D.T. Mourya);; Airport Authority of India, New Delhi (S. Matto)

**Keywords:** Ebola, Filovirus, IgG, real-time RT-PCR, viruses, India, follow-up

**To the Editor:** The 2014–2015 epidemic of Ebola virus disease (EVD) in West Africa affected 23,666 persons and caused 14,603 deaths (*1*). The World Health Organization (WHO) declared the epidemic a public health emergency (*2*). Although Ebola virus is transmitted by unprotected physical contact with infected persons, published reports about which body fluids are infected or the risk for fomite transmission are few (*3*). For most cases, virus was detected by reverse transcription PCR (RT-PCR) of clinical (saliva, feces, semen, breast milk, tears, nasal blood, skin swab) and environmental specimens (*4*). Earlier reports of the follow-up of recovered patients stated that viral RNA was detected by RT-PCR for up to 33 days in vaginal, rectal, and conjunctival swab samples from 1 patient and up to 101 days in seminal fluid from 4 patients. Infectious virus was detected in 1 seminal fluid sample 82 days after disease onset (*4**,**5*).

Attendees at the Eighth Meeting of the WHO Advisory Group on the EVD Response (*1*) discussed potential risk factors, including hidden chains of transmission and sexual transmission, and determined the following criteria. A country can declare “interruption of transmission” when 42 days have elapsed since the last diagnosis of a case. A country can declare that the “outbreak has stopped” when test results from the last case are negative twice or after another 90-day interval. For determining a cutoff for finally declaring the strategy and criteria for elimination, extensive follow-up on infectivity of semen in Ebola survivors is needed.

We report follow-up of a man who recovered from EVD and was monitored for 165 days after he was declared Ebola-free. The 26-year-old man from India returned to New Delhi, India, from Liberia on November 10, 2014, with a certificate from the government of Liberia stating that he was “cured” of Ebola. Because EVD is considered an exotic disease in India, he was placed in isolation at the Airport Health Organization quarantine center at Indira Gandhi International Airport, New Delhi (*6*). Serum and semen samples were collected and sent to the National Centre for Disease Control (NCDC), New Delhi, and the National Institute of Virology (NIV), Pune, India. The serum was negative by real-time RT- PCR ([*7*]; http://www.cdc.gov/vhf/ebola/diagnosis/index.htm) and positive (titer 1:400) by IgG ELISA (*8*). However, the semen was positive for Ebola viral RNA by real-time RT-PCR. A viral RNA extraction kit (QIAGEN, Valencia, CA, USA) was used to extract RNA from samples**. **Real-time RT-PCRs were set up in a Bio-Rad real-time PCR machine (Model C1000 Touch CFX96; Hercules, CA, USA) by using a SuperScript III Platinum One-Step qRT-PCR kit with ROX (Invitrogen, Carlsbad, CA, USA).

As a confirmatory measure, partial nucleoprotein gene (1216 nt), partial viral protein (VP) gene (337 nt), and the intergenic region near the VP gene along with VP polyA tail (383 nt) from the semen sample were amplified by RT-PCR (*9*). These sequences (GenBank accession nos. KT191140–KT191142) showed 100% similarity with *Zaire ebolavirus* isolate EBOV/DML14077/SLe/WesternUrban/20150630. To avoid cross-contamination, we used a γ-inactivated Ebola Reston virus strain as a positive control. The RT-PCRs were set up in a Bio-Rad thermal cycler (C1000) by using SuperScript III One-Step RT-PCR System with Platinum-Taq-DNA-Polymerase (Invitrogen).

Ebola virus isolation attempts were made from the semen sample in VeroE6 cells, Vero CCL81,* Pipistrellus *bat embryo, and BHK-21 cell lines at NIV. No isolate was obtained. Cycle thresholds of 28.5 and 31.5 were observed in Vero CCL81 and Vero E6 cells, respectively, in the first passage by real time RT-PCR, but no virus or cytopathic effect was detected in the subsequent 2 passages (Table).

**Table:** Results of attempted real-time RT-PCR and virus isolation from semen of Ebola virus disease survivor, 2014–2015*

Sample no.	Days declared Ebola-free†	RT-PCR C_t_	Cell lines used for virus isolation	Passage

1			2			3			4	
NCDC‡	NIV§	CPE	C_t_	CPE	C_t_	CPE	C_t_	CPE	C_t_
1	45	17	22.5	Vero CCL81	None	28.5		None	None		None	None		None	None
				Vero E6	None	31.5		None	None		None	None		ND	None
2	64	21	24	Vero CCL81	None	None		None	None		None	None		ND	ND
				Vero E6	None	32.5		None	None		None	None		ND	ND
3¶	77	22	ND	NA	NA	NA		NA	NA		NA	NA		NA	NA
4¶	98	26	ND	NA	NA	NA		NA	NA		NA	NA		NA	NA
5¶	111	27	ND	NA	NA	NA		NA	NA		NA	NA		NA	NA
6	125	30	34	Vero CCL81	None	None		None	None		ND	ND		ND	ND
				Vero E6	None	None		None	None		ND	ND		ND	ND
7	141	28	38	Vero CCL81	None	None		None	None		ND	ND		ND	ND
				Vero E6	None	None		None	None		ND	ND		ND	ND
8	165	28	35.0	Vero CCL81	None	None		None	None		ND	ND		ND	ND
				BHK-21	None	None		None	None		ND	ND		ND	ND

*C_t_, cycle threshold; CPE, cytopathic effect; NA, not applicable; NCDC, National Centre for Disease Control, New Delhi, India; ND, not done; NIV, National Institute of Virology, Pune, India; RT-PCR, reverse transcription PCR.  †Primers from http://www.crcf.sn/wp-content/uploads/2014/09/CDC-Ebola-International-Lab.pdf. ‡Primers from the Centers for Disease Control and Prevention, Atlanta, Georgia, USA. §Primers from (7). ¶Samples received at NCDC only.

According to WHO guidelines, the semen sample was transported from NCDC to a Biosafety Level 4 laboratory at NIV (for virus isolation). At the time of inoculation in cell culture, the sample had been subjected to a single freeze–thaw cycle. The sensitive nature of the virus may be why Ebola virus was not isolated. PCR positivity alone is not sufficient for considering a patient infectious for Ebola; however, because EVD is considered an exotic disease in India, we depended on real-time RT-PCR–based data for establishing EVD positivity.

Follow-up semen samples were positive by real time RT-PCR for up to 165 days after the patient was declared Ebola-free and were negative thereafter. Cycle thresholds of samples tested at NIV were 22.5 on day 45 after being declared Ebola-free, 24 on day 64, 34 on day 125, 38 on day 141, and 35.0 on day 165 (Table). 

Clear criteria for elimination and declaration of the end of an outbreak are needed because any misinterpretation or miscommunication among the countries could negatively affect community confidence (*10*). Although we monitored the patient for 165 days, monitoring began ≈10 days after the patient had recovered. Ebola viral RNA persistence has been documented in a human semen sample for up to 10 months after the patient was declared Ebola-free (*11*). According to the data from this study, the current elimination period may need to be extended, and further studies on the infectivity of semen samples from recovered EVD patients are warranted.
